# Telomerase inhibitors TMPyP4 and thymoquinone decreased cell proliferation and induced cell death in the non-small cell lung cancer cell line LC-HK2, modifying the pattern of focal adhesion

**DOI:** 10.1590/1414-431X2023e12897

**Published:** 2023-10-27

**Authors:** A.M.B. Garnique, P. Rezende-Teixeira, G.M. Machado‐Santelli

**Affiliations:** 1 Departamento de Biologia Celular e do Desenvolvimento, Instituto de Ciências Biomédicas, Universidade de São Paulo, São Paulo, SP, Brasil; 2 Departamento de Farmacologia, Instituto de Ciências Biomédicas, Universidade de São Paulo, São Paulo, SP, Brasil

**Keywords:** TMPyP4, Thymoquinone, Cell viability, Cell adhesion, Telomerase

## Abstract

G‐quadruplexes (G4) are structures formed at the ends of telomeres rich in guanines and stabilized by molecules that bind to specific sites. TMPyP4 and thymoquinone (TQ) are small molecules that bind to G4 and have drawn attention because of their role as telomerase inhibitors. The aim of this study was to evaluate the effects of telomerase inhibitors on cellular proliferation, senescence, and death. Two cell lines, LC‐HK2 (non-small cell lung cancer - NSCLC) and RPE‐1 (hTERT-immortalized), were treated with TMPyP4 (5 μM) and TQ (10 μM). Both inhibitors decreased telomerase activity. TMPyP4 increased the percentage of cells with membrane damage associated with cell death and decreased the frequency of cells in the S‐phase. TMPyP4 reduced cell adhesion ability and modified the pattern of focal adhesion. TQ acted in a concentration-dependent manner, increasing the frequency of senescent cells and inducing cell cycle arrest in G1 phase. Thus, the present results showed that TMPyP4 and TQ, although acting as telomerase inhibitors, had a broader effect on other signaling pathways and processes in cells, differing from each other. However, they act both on malignant and immortalized cells, and further studies are needed before their anti-cancer potential can be considered.

## Introduction

Telomeres are structures at the ends of eukaryotic chromosomes that provide chromosome protection from end fusion and prevent the loss of genetic information. In humans, they are composed of telomere-binding proteins and TTAGGG tandem repeat DNA sequences that have the potential to form a unique G-quadruplex (G4) secondary structure. The length of telomeric DNA determines the cellular replicative capacity of mammalian cells and, except in embryonic germ cells, stem cells, and cancer cells, telomere length gradually shortens with cell division, reaching the “Hayflick limit” and cellular senescence (1). The molecule responsible for maintaining telomeres is telomerase, an enzyme that contains two essential subunits, the protein subunit human telomerase reverse transcriptase (hTERT) and the RNA subunit human telomerase RNA (hTERC or hTER). Cell immortalization in oncogenesis of the majority of cancers is dependent on telomerase reactivation. Telomerase activity is of great significance for the regulation of cell senescence and cell division, and many studies have focused on the regulation of telomerase activity in the hope of developing new strategies to achieve targeted telomerase inhibition for cancer therapy and its activation (2,3).

Telomere elongation and telomerase activity can be inhibited by G4 structures since the telomere terminal repeat sequence TTAGGG is highly enriched in guanine. G4 are structures formed by four chains of guanine-rich sequences; these structures can be stabilized by monovalent cations forming Hoogsteen bonds (4-6). *In vivo* studies have demonstrated the presence of G4 at regulatory regions of genes and at chromosomal ends (telomeres). Different studies have shown control over the expression of oncogenes such as C-MYC, C-KIT, and K-RAS, which can further stimulate recombination in meiosis (5,7-9). However, another important role of G4 formation is the inhibition of telomerase activity. In cancer cells, telomerase is activated, which permits it to bind to the telomere and elongate it, allowing cancer cells to continue to proliferate (4,9).

This characteristic is very important, as almost 90% of the described cancers show telomerase activity, whereas only 10% show another pathway independent of telomerase activity. Therefore, in recent years, different studies have investigated molecules that bind to and help stabilize G4s (4).

Monovalent cations such as Na+ and K+ were identified to help stabilize G4s. Later, studies of G4 interactions with different small molecules n cancer cells in culture were performed, and research on small molecules that bind to G4 has increased (4,5). Molecules that bind to G4 are capable of causing telomere dysfunction and chromosomal instability (8-10). Among these molecules, we highlight TMPyP4, a molecule derived from a cationic porphyrin that stabilizes G4 and causes telomere dysfunction in addition to other deleterious effects in cancer cells (5,8,11). Its potential in the inhibition of telomerase activity has been evaluated because it is a G4 stabilizer in prostate, breast, colon, and other cancer cells (4). In a study conducted by Lin et al. (8), it was demonstrated that the use of TMPyP4 decreases the growth of ovarian cancer cells, causes modification in cell morphology, and increases the number of apoptotic bodies in a concentration-dependent manner.

TMPyP4 can bind and stabilize G4 structures, and it was found through cDNA microarray assays that TMPyP4 also downregulates the expression of the *c-myc* oncogene, which is responsible for regulating the hTERT catalytic subunit of telomerase. Therefore, TMPyP4 can modulate the activity of telomerase by two different pathways (12). In another study with ovarian carcinoma cells, the use of TMPyP4 combined with photodynamic therapy suppressed the growth, proliferation, and motility of cells (7). Cheng et al. (13) showed in a study on normal and cervical carcinoma cells that TMPyP4 at different concentrations was responsible for apoptosis induction and decreased proliferation in cancer cells by activation of the p38 MAPK signaling pathway.

Similarly, thymoquinone (TQ), a component derived from the seed of *Nigella sativa* Linn, has been reported as an antitumor agent using different mechanisms, including associating with telomeric DNA by stabilizing G4 to inhibit telomerase activity and inhibiting proliferation in cancer cells (14). *In vivo* studies have shown the antitumor activity of TQ treatment, which results in DNA damage, telomere shortening, cell cycle arrest, and apoptosis in cancer cells and in cells immortalized with hTERT (15). Other effects of TQ include oxidative damage and inhibition of angiogenesis, migration, invasion, and metastasis of cancer cells (16,17).

A wide range of responses have been observed in studies investigating the effects of TQ and TMPyP4 on cancer cells in both *in vivo* and *in vitro* settings. However, there is limited knowledge regarding their specific effects in lung cancer, while most research has focused on breast cancer. The elucidation of their effects on cancer cells is of fundamental importance for understanding the action of G4 stabilizing compounds and evaluating their potential as anticancer agents.

The present study aimed to compare the effects of two G4 stabilizing compounds in non-small human lung cancer cells (LC-HK2) and an hTERT-immortalized cell line (RPE-1).

## Material and Methods

### Cell lines

The LC-HK2 cell line from a human non-small cell lung carcinoma established in our laboratory (18,19) and the commercial line hTERT-RPE-1, epithelial cells immortalized with hTERT from human retinal pigment epithelial cells, were grown in Dulbecco's modified Eagle's minimal medium (DMEM) with F-12 nutrient from Sigma (USA), supplemented with 10% fetal bovine serum (Cultilab, Brazil). Cultures were kept at 37°C, with the atmosphere containing ∼5% CO_2_. The culture medium was changed every two to three days and the cells were subcultured regularly. Subcultures were obtained by dissociation with 0.05% trypsin solution and 0.02% EDTA.

### Inhibitors

Telomerase inhibitors TMPyP4 (Calbiochem, Merck, Germany, CAS number: 36951-72-1, stock solution 1 mg/mL) and thymoquinone (TQ) (Sigma, CAS number: 490-91-5, stock solution) were used. The TMPyP4 inhibitor was used at a concentration of 5 μM, a value obtained from previous experiments in our laboratory (data not shown), and TQ at concentrations of 10 μM, a value obtained from trypan blue cell viability assays. The cell lines were treated for 72 and 144 h at the above concentrations, and control samples were without inhibitors for the same period.

### Trypan blue viability assay

The two cell lines were seeded, and after 24 h the cells were treated with the TQ inhibitor at concentrations of 10-100 μM. After 24 h, the cells were resuspended by trypsin dissociation and trypan blue at 0.4% 1:1 was added for 3 min. Then, the cells were counted in the Neubauer chamber (Electron Microscopy Sciences, USA).

### Cell cycle analysis by PI staining

After treatments, cells were resuspended using trypsin-EDTA, centrifuged at 300 *g* for 10 min at 25°C, washed with phosphate buffered saline A (PBSA, without Ca^+^ and Mg^+^), and centrifuged again (300 *g*, 10 min, 25°C). Samples were fixed with 75% methanol at 4°C for 1 h and washed with PBSA. DNA was stained with propidium iodide (PI) (10 μg/mL) and treated with RNase (10 µg/mL) at 4°C for 1 h and quantified by a flow cytometer (GUAVA EasyCyte Plus, USA). The assay was conducted three times in replicates, and the results are reported as means±SD of the percentage of cell distribution in cell cycle phases (G1, S, and G2/M).

### Senescence-associated **β**-galactosidase staining

The senescence assay by β-galactosidase staining was performed by a protocol modified from Campisi (20) and Itahana et al. (21). The cells were fixed with a fresh solution of 3.7% formaldehyde (Sigma-Aldrich, USA) for 20 min at room temperature, washed with PBSA, and then added to the staining solution of 1 mg/mL of X-gal dissolved in dimethylformamide, 40 mM citric acid/sodium phosphate buffer (pH 6.0), 5 mM potassium ferrocyanide solution, 5 mM potassium ferricyanide solution, 150 mM of NaCl, and 2 mM of MgCl_2_. The plate was sealed with parafilm and incubated overnight at 37°C. Coverslips were mounted on microscope slides with 70% glycerol in PBSA. After this, the blue-stained cells were counted.

### Immunofluorescence and fluorescence assay

For immunostaining, samples were fixed with 3.7% formaldehyde (Sigma-Aldrich) for 30 min, permeated with Triton X-100 (0.5%) for 30 min, and incubated overnight with primary monoclonal antibodies mouse anti-α-Tubulin (1:50, Cell Signaling Technology 3873, USA) and mouse anti-β-Tubulin (1:50, Cell Signaling Technology 86298) and then 2 h with secondary antibodies anti-mouse IgG (H+L) and F (ab')2 Fragment (Alexa Fluor^®^ 555 or 488 Conjugate, Thermo Fisher Scientific, USA) (1:50). Nuclei were labeled with DAPI (1:100) or PI (10 mg/mL) from Sigma-Aldrich. The slides were mounted with Vecta-Shield from Vector Laboratories (USA) and analyzed by laser scanning confocal microscopy from Leica TCS SP8 (Germany).

### Mitotic index

Mitotic index was calculated by manual counting of the number of mitoses/1000 cells per sample from fluorescent cytological preparations stained for microtubules and nuclei. Images were obtained using the Axioscan Z1 slide scanner microscope (Carl Zeiss Microscopy GmbH, Germany) and processed by ZEN blue edition software (https://www.zeiss.com). The results are reported as means±SD of the percentage of cells in mitoses/total number of counted cells of three independent experiments.

### EdU incorporation

The frequency of S-phase cells was determined by the incorporation of EdU (5-ethynyl-2'-deoxyuridine) using Click-iT^®^ EdU Imaging kit (Invitrogen, USA) following the manufacturer's instructions.

### Telomerase activity assay

Quantitative determination of telomerase activity was performed according to the Telomeric Repeat Amplification Protocol using the TeloTAGGG Telomerase PCR ELISAPLUS kit (Roche Diagnostic GmbH, Germany). After treatment with the inhibitors, LC-HK2 and RPE-1 cell lines were lysed using 2 mL lysis buffer and centrifuged at 4°C for 30 min at 20,000 *g*. The supernatants of different samples were divided into two aliquots before performing the assay: one was used as negative control after heat inactivation of telomerase at 80°C for 10 min and the other was used to evaluate the telomerase-mediated addition of the telomeric sequence. The products obtained were amplified by PCR. To exclude false-negative results due to Taq DNA-polymerase inhibitors eventually present in the lysates, a 216-bp long internal standard, present in the reaction mixture, was simultaneously amplified. The results are reported as relative telomerase activity (RTA), which was calculated according to the following formula: RTA = [(Abs sample - Abs RNAse-treated sample) / Abs internal standard of the sample] / [(Abs control template - Abs lysis buffer) / Abs internal standard of the control template] × 100, where Abs = absorbance.

### Cell adhesion assay

After the treatment, the adhesion potential was analyzed using the washing adhesion assay. The cells were plated and, after treatment with the inhibitors, cells were resuspended with trypsin and placed on a 24-well plate containing collagen A in the bottom. After approximately 4-h incubation, the cells were washed three times with PBSA, and the adherent cells were fixed and stained with violet crystal. The number of adherent cells was obtained from three independent experiments.

### Real time PCR assay

Total RNA from control and treated groups of LC-HK2 and RPE-1 cell lines was extracted using the ChargeSwitch Total RNA Cell Kit (Invitrogen) and quantified on a NanoDrop ND1000 spectrophotometer (Thermo Fisher Scientific). Expression profile analysis was performed by real-time RT-PCR reactions on a Corbett Research model Rotor Gene 6000 real-time cycler and the AgPath-ID One-Step RT-PCR kit (Applied Biosystems, USA). Real-time PCR conditions were 45°C for 10 min; 95°C for 10 min, 40 cycles (95°C for 15 s; 60°C for 45 s), followed by melting curve analysis. The primers used were: qVinc1 GCACCCAGCTCAAAATCCTG; qVinc2 TCAGCCAGGTGCCTACTGGT; qVim-left GAGAACTTTGCCGTTGAAGC; and qVim-right TCCAGCAGCTTCCTGTAGGT, which amplify fragments of up to 250 bases; normalization was done by the total RNA mass used by each reaction, as suggested by Bustin (22,23).

### Zombie Green assay

Cells with membrane damage, which may have been caused by inhibitor treatments, were evaluated by the Zombie Green assay using the Zombie Green™ Fixable Viability Kit (Biolegend, USA). Cells were treated or not with the inhibitors following the manufacturer's instructions.

### Statistical analysis

Results are reported as means±SD. Data were analyzed by ANOVA, followed by Dunnet's test for multiple comparisons with the control. A P-value <0.05 was considered statistically significant.

## Results

### Cell viability assay for thymoquinone

**Figure 1:**
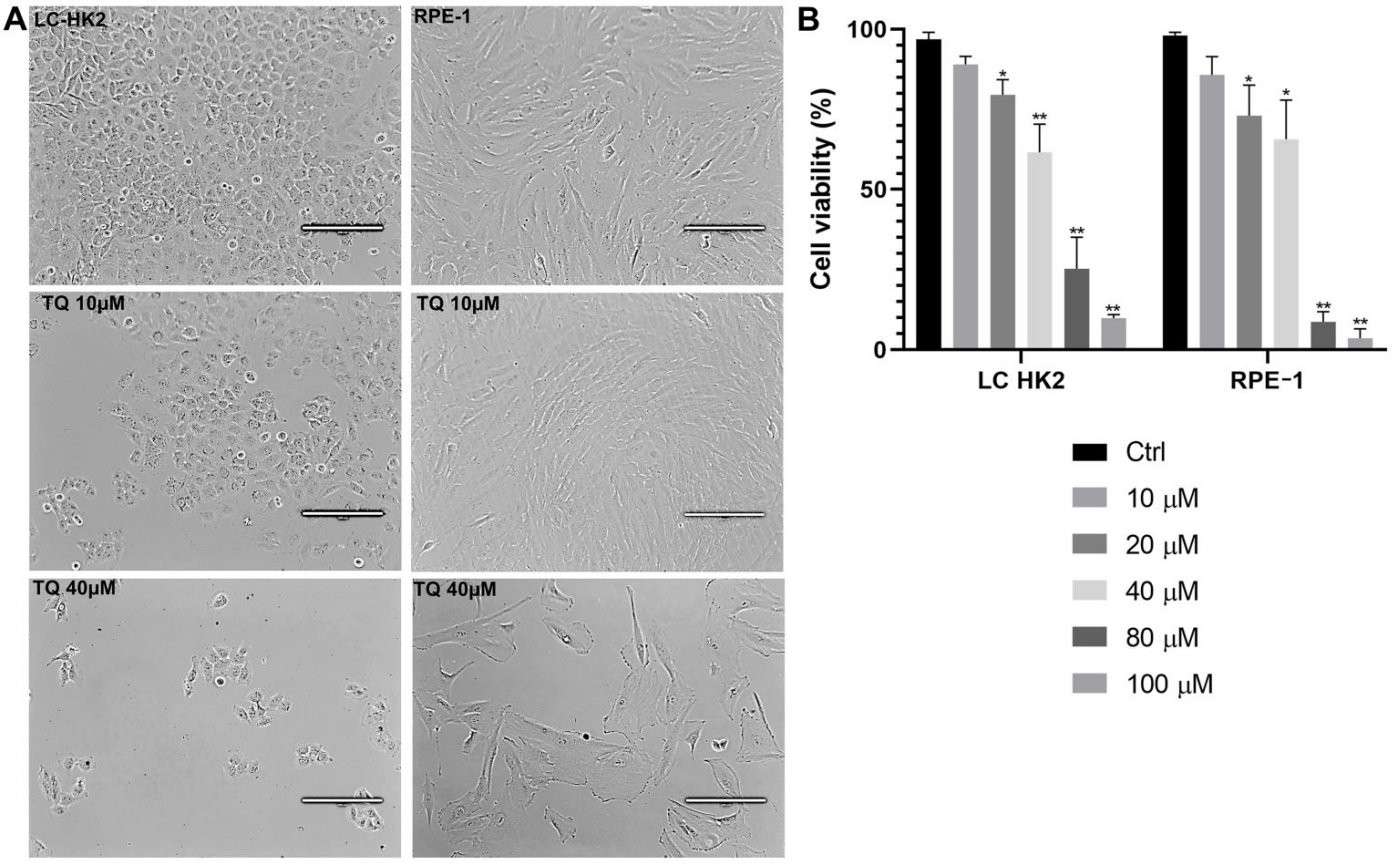
Cell viability assay of thymoquinone (TQ)-treated LC-HK2 and RPE-1 cell lines: **A**, Phase contrast image of LC-HK2 and RPE-1 cell lines in control and TQ treatment (10 and 40 μM) conditions. Scale bar: 200 μm. **B**, Both cell lines were treated with TQ at concentrations of 10-100 μM for 24 h. Data are reported as mean±SD percentage of the control in three independent experiments. *P<0.05, **P<0.01, ANOVA and Dunnett's test for multiple comparisons *vs* control.

TQ showed antitumor potential by inhibiting cell proliferation and inducing apoptosis (24), and new studies have shown an association with the formation of G4. Thus, the cell viability assay was performed using trypan blue staining. The LC-HK2 cell line showed a decrease in cell viability compared to the control; at concentrations of 20, 40, 80, and 100 μM, the cell viability was 79.56, 61.65, 25.31, and 9.87% of viable cells, respectively (Figure 1).

In the RPE-1 cell line, this reduction was more accentuated at the highest concentrations; the cell viability in the control was 98% while that of the cells treated with 20, 40, 80, and 100 μM TQ was 73, 65.66, 8.73, and 3.63%, respectively (Figure 1). Therefore, this inhibitor showed a concentration-dependent response on cell viability in both cell lines.

### TMPyP4 and TQ inhibited telomerase activity

**Figure 2:**
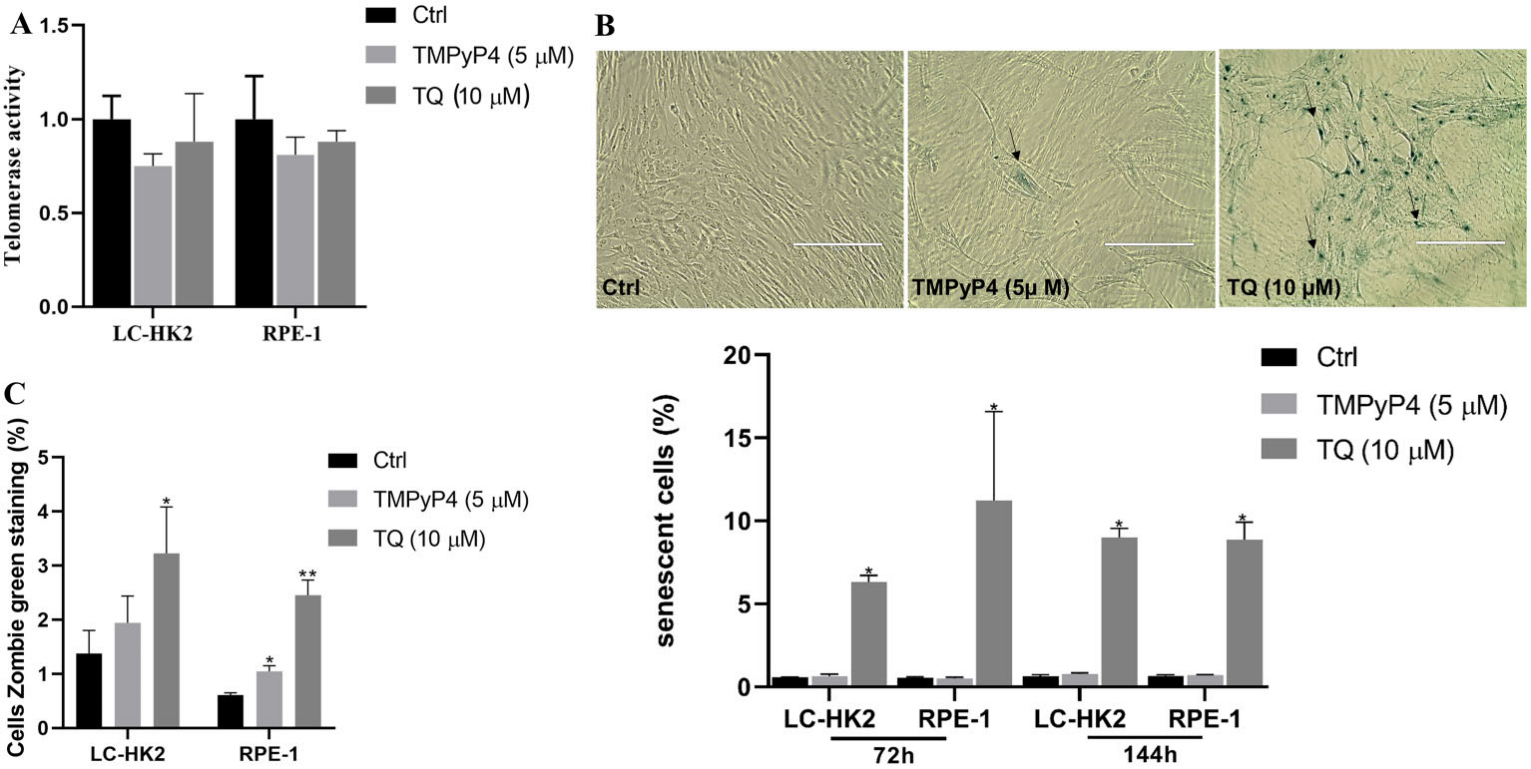
**A**, Evaluation of telomerase activity in LC-HK2 and RPE-1 cells treated with TMPyP4 (5 μM) and thymoquinone (TQ) (10 μM) inhibitors for 72 h to evaluate telomerase activity. Data are reported as mean±SD of two independent experiments. **B**, Photomicrography of senescence staining assay on RPE-1 cells; arrows indicate senescent cells. Scale bar: 200 μm. The graph below the images shows the mean±SD percentage of senescent cells treated with telomerase inhibitors compared to control in three independent experiments. **C**, Percentage of membrane damage of cells treated with telomerase inhibitors. Data are reported as mean±SD in three independent experiments. *P<0.05, **P<0.01, ANOVA and Dunnett's test for multiple comparisons *vs* control.

Treatment with TMPyP4 (5 µM) for 72 h reduced telomerase activity in LC-HK2 and RPE-1 cells, with values of 0.74 and 0.81, respectively, compared to control cells (Figure 2A). Cells treated with TQ (10 µM) also showed decreased telomerase activity; in LC-HK2 cells this value was 0.88, and in RPE-1 cells this value was 0.87 (Figure 2A). Various spectroscopic techniques, including circular dichroism, have been used to demonstrate that TMPyP4 and TQ are able to bind and stabilize G4s in telomeres, thus inhibiting the telomerase enzyme binding and decreasing the activity of the enzyme (14,25).

One of the characteristics of senescent cells is the induction of the expression of senescence-associated β-galactosidase (SA-β-Gal), in addition to a flat morphology (26). The LC-HK2 and RPE-1 cell lines treated with TMPyP4 (5 µM) for 72 h showed frequencies of senescent cells of 0.63 and 0.5%, respectively. The control cells showed a frequency of senescence of 0.57% in LC-HK2 and 0.54% in RPE-1 in the same treatment period (Figure 2B). After 144 h, it was observed that TMPyP4 does not induce senescent cells (Figure 2B).

For the induction of cellular senescence by TQ, the concentration used in the cell treatments was 10 µM, a non-lethal concentration, and the treatments were performed for 72 and 144 h. TQ (10 µM) induced an increase in the percentage of senescent cells. In LC-HK2 cells, the percentage of senescent cells was 6.3% (72 h) and 8.9% (144 h). The RPE-1 cells showed a high percentage of senescent cells of 11.23% at 72 h and 8.87% at 144 h (Figure 2B). A reduction in cellular density was observed in the cells treated with TQ; thus, cell death was evaluated by the Zombie Green assay. This assay marks cells that have suffered damage to the cell membrane and subsequently undergo cell death.

The LC-HK2 cells treated with TMPyP4 had membrane damage in 1.94% of the cells compared to 1.37% in the control cells, and when treated with TQ (10 µM) this percentage was 3.22% at 72 h (Figure 2C). In RPE-1 cells, 0.61% of the control cells showed cell membrane damage, compared to 1.04% of the cells treated with TMPyP4 (5 µM). Treatment with TQ (10 µM) resulted in membrane damage in 2.45% of the cells after 72 h, indicating an increase in cells with membrane damage, which undergo cell death induced by treatments with the inhibitors (Figure 2C).

### TMPyP4 and TQ modified cell proliferation

**Figure 3:**
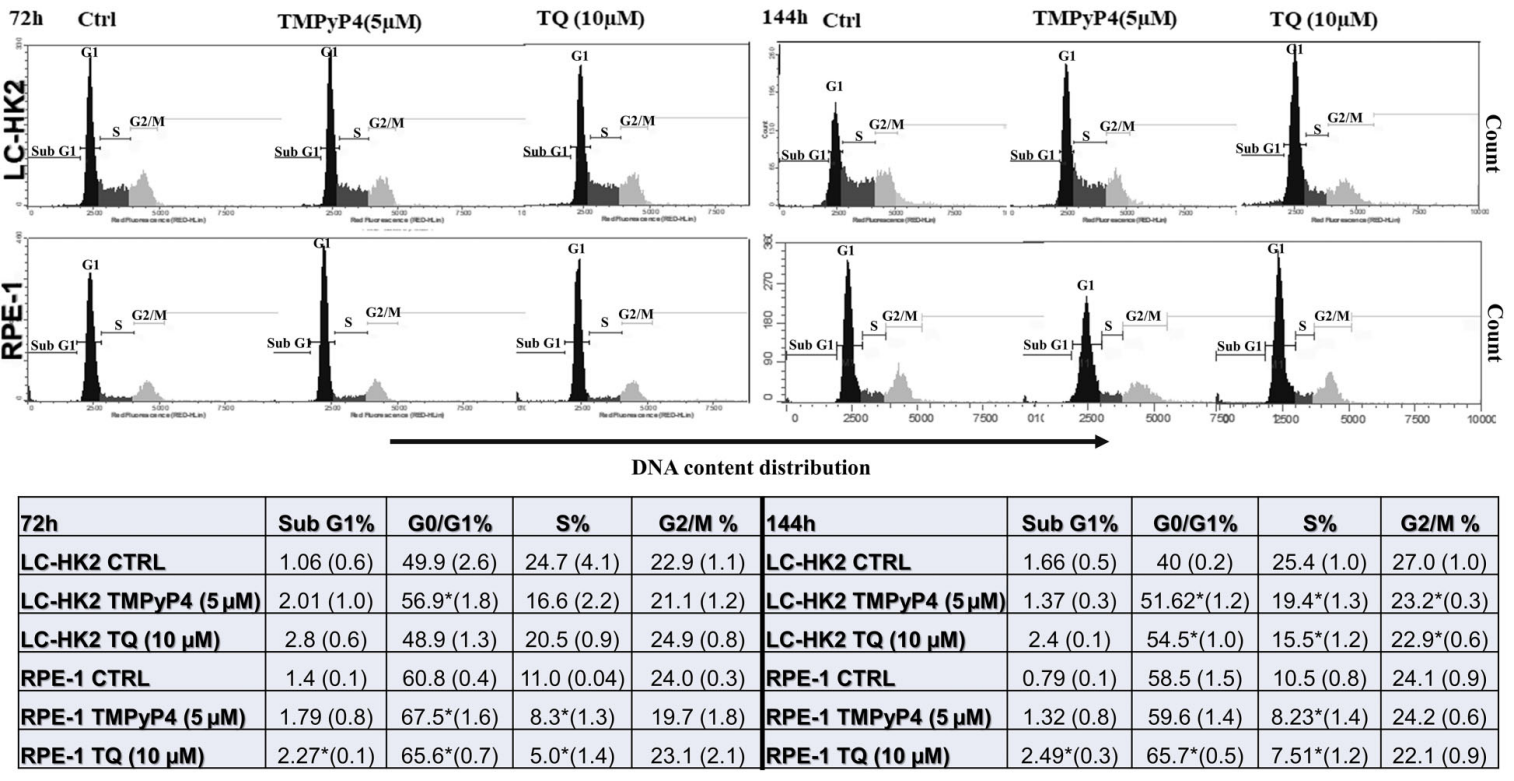
Distribution of cell cycle phases. LC-HK2 and RPE-1 cells treated with TMPyP4 (5 µM) and thymoquinone (TQ) (10 µM) for 72 and 144 h showed increased percentages in some phases of the cell cycle, as well as a difference in response to treatment depending on the cell line. Data are reported as mean±SD percentage in three independent experiments. *P<0.05, ANOVA and Dunnett's test for multiple comparisons *vs* control.

Cell proliferation was evaluated by cell cycle assays, EdU incorporation, and the mitotic index. For cell cycle profile evaluation, cells were treated with the inhibitors and then stained with PI. Cells treated with TMPyP4 (5 µM) showed differences in cell cycle phases (Figure 3). After 72 h of treatment, an increase in the G0/G1 phase cell population was observed compared to control cells (49.9% for LC-HK2 cells and 60.8% for RPE-1 cells), and the frequency observed was 56.9% for LC-HK2 cells and 67.65% for RPE-1 cells. The cell population in the S phase was 16.66% for LC-HK2 cells and 8.3% for RPE-1 cells, which showed a decrease compared to control cells (24.7% for LC-HK2 cells and 11% for RPE-1 cells). After 144 h of treatment, the LC-HK2 cells showed an increase in the cell population in the G0/G1 phase (51.62%) compared to the control (40%) and a decrease in populations in the S phase (19.43%) and G2/M phase (23.24%). RPE-1 cells showed a decrease in the S phase cell population (8.23%) and G2/M phase (24.2%) (Figure 3).

After TQ treatment for 72 h, LC-HK2 cells showed a decrease in the S phase cell population (20.51%). In RPE-1 cells treated with TQ (10 µM), there was a decrease in the S phase cell population (5%) and an increase in the sub-G1 phase (2.27%), G1 phase (65.67%), and G2/M phase (26.27%). In analyses of the treatments with TQ (10 µM) for 144 h, LC-HK2 cells showed an increase in the G1 phase cell population (54.52%) and a decrease in S (15.55%) and G2/M (22.94%) phases. The RPE-1 cells showed an increase in sub-G1 (2.49%) and G0/G1 (65.78%) phases, with a decrease in S (7.51%) phase (Figure 3).

### Mitotic index and EdU assay

**Figure 4:**
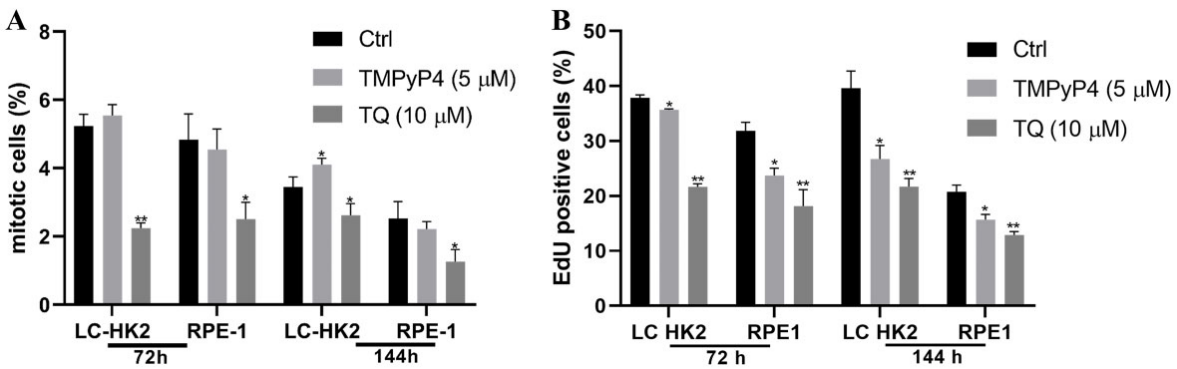
A, A decrease of mitotic cells was observed after treatment with the inhibitors. **B**, Percentage of EdU-labeled cells. A decrease in EdU-positive cells was observed after treatment with the inhibitors. Data are reported as mean±SD in three independent experiments. *P<0.05 and **P<0.01, ANOVA and Dunnett's test for multiple comparisons *vs* control.

In the study, the effects of TQ and TMPyP4 on the mitotic index were assessed at different time points of treatment. The treatment with TMPyP4 (5 µM) for 72 h in the LC-HK2 cell line resulted in a mitotic cell percentage of 5.54%, compared to 5.23% in the control cells. Similarly, in the RPE-1 cell line, the percentages were 4.55 and 4.83% for the treated and control cells, respectively. After 144 hours of treatment, the LC-HK2 cell line exhibited a mitotic cell percentage of 4.1%, while the control cells showed a percentage of 3.44%. In the RPE-1 cell line, the treated cells displayed a percentage of 2.21% compared to 2.53% in the control cells (Figure 4A).

The treatment with TQ (10 µM) for 72 h yielded a mitotic cell percentage of 2.24% in the LC-HK2 cell line and 2.51% in the RPE-1 cell line. After 144 h, the mitotic cell percentage in the LC-HK2 cell line was 2.62%, while in the RPE-1 cell line it was 1.26% (Figure 4A).

An EdU assay was performed to determine the number of cells in the S phase of the cell cycle. After treatment with the inhibitors, a decrease in the number of cells in the DNA synthesis phase was observed. The LC-HK2 and RPE-1 cell lines had 37.86 and 31.86% (of control) of cells in the S phase, respectively. After treatment with TMPyP4 (5 µM) for 72 h, 35.66% of the LC-HK2 cells and 23.74% of the RPE-1 cells were in the S phase. After treatment with TQ (10 µM), 21.63% of the LC-HK2 cells and 18.15% of RPE-1 cells were found to be in the S phase (Figure 4B).

After 144 h of treatment, the reduction in the percentage of cells in the S phase was sustained, with LC-HK2 and RPE-1 control cells showing percentages of 39.61 and 20.72%, respectively. Treatment with TMPyP4 (5 µM) resulted in 26.73% of LC-HK2 cells and 15.68% of RPE-1 cells in the S phase, and finally, treatment with TQ (10 µM) resulted in 21.67% of LC-HK2 cells and 12.9% of RPE-1 cells in the S phase (Figure 4B).

### TMPyP4 caused alterations in the actin cytoskeleton and cell adhesion

**Figure 5:**
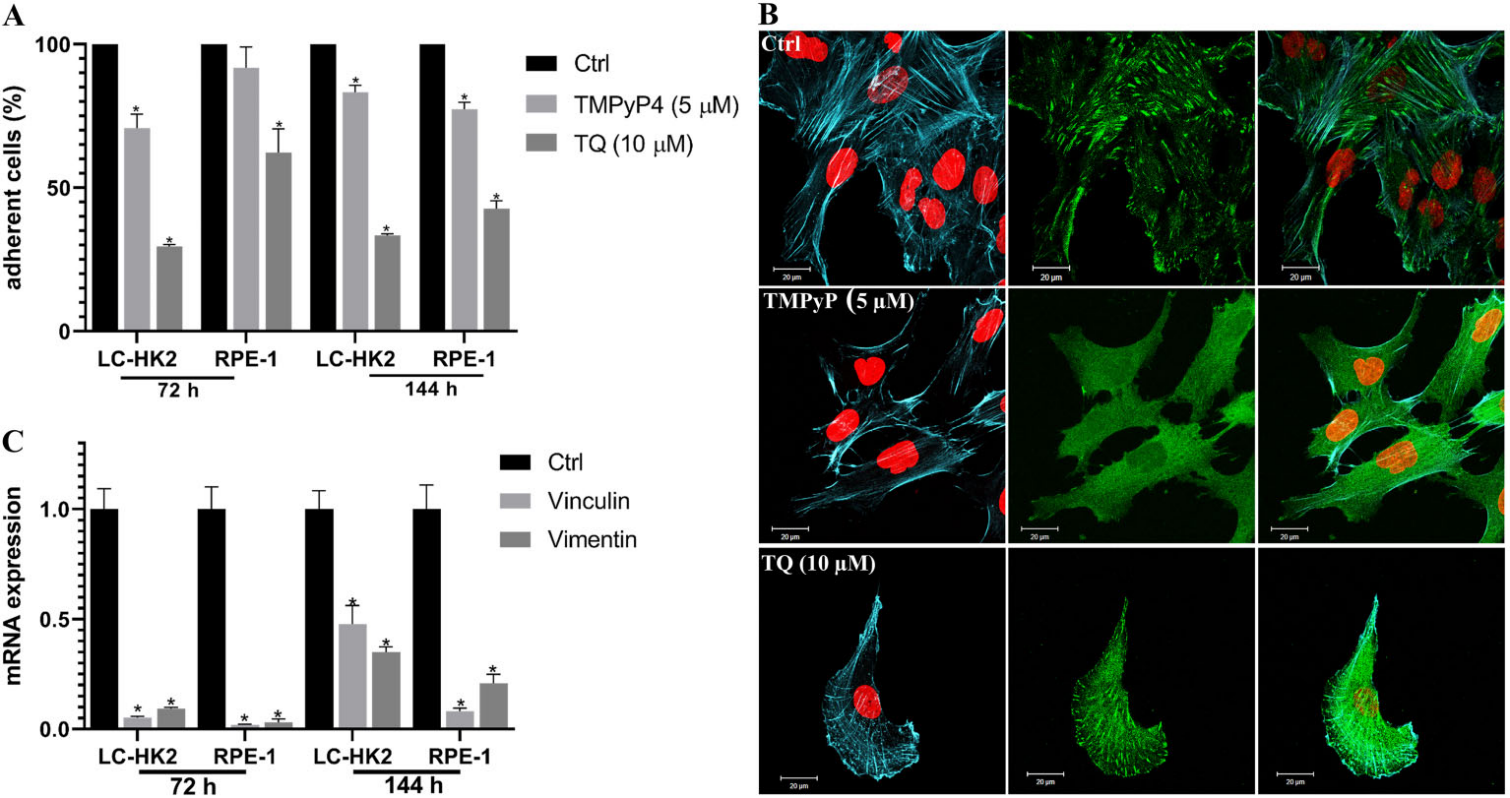
A, Percentage of adherent cells after treatment with inhibitors. **B**, Immunofluorescence of focal adhesions formed by vinculin protein: Cells treated for 72 h with the TMPyP4 inhibitor did not show the focal adhesion (green) formed by vinculin, whereas it is seen in control and cells treated with thymoquinone (TQ) (10 µM). Nuclei are shown in red and actin cytoskeletons in blue. Scale bar: 20 µm. **C**, Expression of vinculin and vimentin mRNA in TMPyP4 treated cells. Data are reported as mean±SD. *P<0.01, ANOVA and Dunnett's test for multiple comparisons *vs* control.

Treatments with TMPyP4 (5 µM) led to a decrease in surface-adhered LC-HK2 cells. After 72 h, 70.78% of the cells adhered, and after 144 h, 83.26% of the cells adhered. For RPE-1 cells, treatment with TMPyP4 (5 µM) resulted in 91.8% of adhered cells after 72 h, and after 144 h, 77.3% of the cells remained adhered. In cells treated with TQ (10 µM), there was a decrease in the number of adherent cells; after 72 h, 29.53% of the LC-HK2 cells and 62.32% of the RPE-1 cells were adhered. After 144 h, 33.35% of the LC-HK2 cells and 42.7% of the RPE-1 cells were adhered (Figure 5A).

Immunofluorescent staining of the vinculin protein revealed focal adhesion structures in RPE-1 control cells and cells treated with TQ (10 µM). However, when analyzing cells treated with TMPyP4 (5 µM), the formation of focal adhesion structures was not observed (Figure 5B). Additionally, the actin cytoskeleton was stained, and it was observed that this structure followed the focal adhesions of control cells, but this was not observed in the cells treated with TMPyP4 (5 µM) (Figure 5B, the actin cytoskeleton is shown in blue).

Then, the expression of vinculin and vimentin mRNA in the RPE-1 cells treated withTMPyP4 (5 µM) was analyzed, both of which have an association with the process of cellular surface adherence. Downregulation of these mRNAs was already observed in RPE-1 cell line after 72 h, and the downregulation persisted until 144 h of treatment (Figure 5C).

## Discussion

### TQ and TMPyP4 decreased telomerase activity

RPE-1 cells were immortalized by adding the catalytic region of the telomerase enzyme, hTERT, providing telomerase activity similar to that found in tumor cells. Furthermore, RPE-1 cells do not demonstrate another mechanism that can lead to abnormal proliferation of the cell and RPE-1 immortalized cells do not have a phenotype associated with malignancy (27), making them a good tool for the study of telomerase inhibitors.

Both TMPyP4 and TQ are known as telomerase inhibitors that generate and stabilize G4 (14). The telomerase activity assay showed that TQ and TMPyP4 may cause a decrease in telomerase activity in both cell lines tested (LC-HK2 and RPE-1).

**Figure 6:**
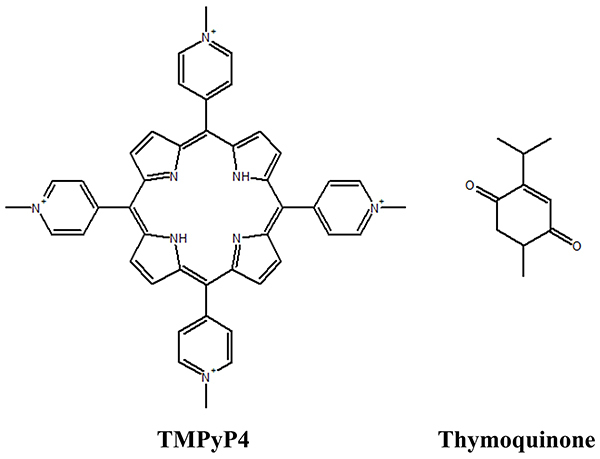
Molecular structures of TMPyP4 and thymoquinone.

TMPyP4 is a cationic porphyrin (Figure 6) with a flat porphine chromophore and four pyridyl rings substituted at the meso positions and binds in the telomeric sequence involving π-π stacking with external loop bases (28,29). TQ is a small molecule that belongs to p-benzoquinones class (Figure 6) and binds to G4 near the loop through π-π stacking intercalation (14).

It has been shown that TQ has anti-proliferative effects on several cancer cell lines, such as ovarian, colon, pancreas, larynx, breast, myeloid leukemia, prostate, lung, and osteosarcoma (30). TQ induces cell cycle arrest, apoptosis, and oxidative damage, blocks angiogenesis, and suppresses migration, invasion, and metastasis (14,15). TMPyP4 also has effects in inducing apoptosis, decreasing cell proliferation, cell cycle arrest, and oxidative damage, but it also acts in the regulation of cell adhesion and migration (7,31).

In this study, both compounds showed inhibition of telomerase activity, inhibition of cell proliferation, as well as cell cycle arrest. However, each compound also showed unique effects. TQ increased the number of senescent cells and cells with cell membrane damage and TMPyP4 decreased cell adhesion probably by decreasing vinculin expression.

### TQ decreased cell viability and induced senescence and cell cycle arrest

Treatment with increasing concentrations of TQ (10-100 µM) reduced cellular viability in a time-dependent manner in both cell lines. An increase in the number of cells with membrane damage may lead to cell death. Similar studies reported this type of death as apoptosis mediated by Bax and Bcl2 signaling (32,33).

TQ treatment of normal cells did not affect cellular viability (34) in contrast to the results of TQ treatment of RPE-1 cell line, which is also a non-tumor cell line, but is immortalized with hTERT, which results in telomerase activity. Thus, this decrease in cell viability may be the result of the association of TQ binding to G4.

The increase in the number of senescent cells in the LC-HK2 and RPE-1 cell lines may be a result of telomere dysfunction, as demonstrated with TQ-treated glioblastoma cells (15). Stochastic telomere attrition or genotoxic stress may lead to dysfunctional telomeres, inducing cellular senescence and assisting in the inhibition of tumor progression (35,36).

The observed arrest in the G1 phase and decrease in the S phase caused by TQ treatment was similar in both cell lines. This effect was also described in hepatocellular carcinoma, where TQ caused arrest in the G1 phase with a consequent decrease in the number of cells in the S phase. Therefore, it was observed that TQ activity depends on the concentration and exposure time of cells, as it can cause senescence or death by signaling proapoptotic proteins (33).

### TMPyP4 modified cellular adhesion

The formation of G4 can affect the binding of transcription factors to nearby sites, which may result in an additional effect on gene transcription (37). G-quadruplex intrachain and G-quadruplex-forming sequences may coincide with promoter regions and play a role in transcriptional regulation or be found at replication origin sites. In addition, G4 was observed to form preferentially in guanine-rich regions (38).

The TMPyP4 inhibitor at a concentration of 5 µM led to a decrease in telomerase activity and changes in the cell cycle, without causing a high level of damage to cell viability at the studied times. However, changes in cell adhesion to the substrate were observed as demonstrated by the cell adhesion assay.

Zheng et al. (31) demonstrated through RNA-seq analysis of the whole genome of the A549 cell line that TMPyP4 can alter gene expression; these changes were found to be positive or negative depending on the concentration of this compound, and the genes found to be affected were associated with adhesion, migration, and cell death. Therefore, proteins associated with cell adhesion and motility, such as vinculin and vimentin, were analyzed. As demonstrated by immunofluorescence staining and mRNA expression assays, this expression was affected in cells treated with TMPyP4.

Vinculin is a protein that participates in the formation of focal adhesions, playing a role in the motility and organization of the actin cytoskeleton and exerting a direct influence on cell migration. Downregulation of vinculin levels has been associated with reduced adhesion and improved cell motility (39).

In this study, a decreased expression of vinculin mRNA levels accompanied by a decrease in vimentin mRNA was observed. Peng et al. silenced vimentin and observed that vimentin and actin cytoskeleton remodeling are required for docking and detachment after cell migration and that this interaction leads to improved tumor cell invasion (40). In addition, a decrease in the levels of vinculin and vimentin was observed, which could be helpful in cell detachment. Later, the levels of vimentin began to increase, and together with the formation of stress fibers, the migration control was resumed; this effect was observed in the first days of TMPyP4 treatment.

### Conclusions

This study provided insights into the differential effects of telomerase inhibitors TMPyP4 and TQ on the LC-HK2 and RPE-1 cell lines at the tested concentrations. Both cell lines exhibited decreased telomerase activity after treatment with these inhibitors. Notably, both inhibitors impacted cell proliferation. TQ treatment resulted in an increase in senescent cells and a decrease in cell viability due to the induction of cell membrane damage, which could lead to subsequent cell death. Conversely, TMPyP4 treatment led to minimal cell membrane damage, with no observed induction of senescence. Additionally, TMPyP4 downregulated the mRNA expression levels of vinculin and vimentin, suggesting a disruption of cell adhesion in the treated cells. These findings contribute to our understanding of the distinct effects of TMPyP4 and TQ, providing valuable insight into their mechanisms of action and their potential implications for cellular behavior. In conclusion, the present results showed that TMPyP4 and TQ, although acting as telomerase inhibitors, have a broader effect on other signaling pathways and cell processes, differing from each other. However, they act both on malignant and immortalized cells and thus more studies are needed so that their anti-cancer potential can be considered.
